# Nurses' Knowledge and Anxiety About Human Monkeypox Virus Infection: A Cross‐Sectional Study

**DOI:** 10.1111/nhs.70162

**Published:** 2025-06-19

**Authors:** Yasemin Karacan, Rıdvan Bayram, Serkan Budak

**Affiliations:** ^1^ Nursing Department, Internal Medicine Nursing Yalova University Faculty of Health Sciences Yalova Türkiye; ^2^ Internal Medicine Nursing Bursa Uludag University Faculty of Health Sciences Bursa Türkiye; ^3^ Department of Health Care Services Kütahya Health Sciences University, Simav Vocational School of Health Services Kütahya Türkiye

**Keywords:** anxiety, disease outbreaks, knowledge, Monkeypox, nurses

## Abstract

This study evaluated the knowledge and anxiety levels of nurses in Türkiye regarding human monkeypox virus (Mpox) infection and explored related factors such as institutional training and preparedness. A descriptive, cross‐sectional study was conducted between October 2024 and January 2025 with 214 actively employed nurses. Data were collected using a socio‐demographic questionnaire, a nurse introduction form, a form assessing thoughts about Mpox, a knowledge assessment form, and the Epidemic Anxiety Scale (EAS). The mean epidemic anxiety score was 48.36 ± 19.26, with significantly higher anxiety levels among female nurses. Greater Mpox knowledge was associated with higher job satisfaction, access to institutional information, and participation in training. Fear of infection, fear of transmitting the virus, and fear of losing loved ones significantly influenced knowledge levels. Each unit increase in perceived information adequacy was associated with a 6.56‐point rise in knowledge scores. The findings reveal a substantial knowledge gap about Mpox among nurses in Türkiye, which may hinder outbreak preparedness. Strengthening educational initiatives and integrating epidemic management into nursing curricula are essential to improving nurses' readiness and protecting public health.

**Summary**

- Mpox presents a significant health threat, particularly among children, due to its high rates of complications and mortality, with transmission primarily occurring through direct contact with body fluids, skin lesions, and contaminated materials.
- Although only a single confirmed case of Mpox has been reported in Türkiye, the country remains at substantial risk for future outbreaks due to extensive international travel, tourism, and student mobility.
- Assessing nurses' knowledge and concerns regarding Mpox is critical for designing targeted training programs and strengthening nurses' capacity for epidemic preparedness and response.

## 1 Background

The Monkeypox virus (Mpox) and the Smallpox virus belong to the same family, Poxviridae (Mohamed et al. 2024; Rony et al. 2023). The first human case of Mpox was reported in the Democratic Republic of the Congo in 1970, and the disease is contagious and rarely fatal (Rony et al. 2023). There are two distinct clades of the virus: Clade I, responsible for the increase in cases in Central and East Africa, and Clade II, associated with the global health problem of the Congo Basin/West Africa lineage. The World Health Organization (WHO) declared Mpox a Public Health Emergency of International Concern (PHEIC) in 2022. Clade I has been associated with higher mortality rates compared to Clade II. West African countries are endemic to Clade II, while Central African countries are endemic to Clade I (CDC 2024). In addition, Mpox was reported by the WHO with 28 suspected cases across 12 non‐endemic countries (Khan et al. 2023; Khattab et al. 2024). The virus subsequently spread to more than 60 countries, raising substantial global public health concerns (Rony et al. 2023; Walsh‐Buhi et al. 2024). Between 1 January 2022 and 30 June 2024, a total of 99 176 laboratory‐confirmed cases and 208 deaths were reported to the WHO from 116 countries (T.C. Health Minister n.d.). In the Democratic Republic of the Congo, 43 862 suspected and confirmed cases were recorded in 2024. In the United States, an average of seven cases per week was observed in January 2024, which decreased to approximately one case per week by November 2024 (CDC 2024). In Türkiye, the Minister of Health announced on 30 June 2022 that Mpox was detected in a patient with an immunocompromised condition (BBC 2022). Although only one case has been officially reported in Türkiye, the potential risk remains significant due to factors such as international tourism and student mobility.

In children infected with Mpox, the likelihood of developing complications and mortality is higher compared to adults (Mohamed et al. 2024; Rony et al. 2023). Although Mpox infection in humans is unrelated to chickenpox, the symptoms are clinically similar, including chills, fever, headache, lymphadenopathy, muscle aches, fatigue, and rash (Khan et al. 2023; Khattab et al. 2024; Rony et al. 2023). The virus can be transmitted from infected animals to humans, or between humans, through direct contact with body fluids, skin lesions, or scabs (CDC 2024). Transmission can also occur through close physical contact such as kissing, hugging, or touching contaminated clothing or bedding (Rony et al. 2023). Additionally, vertical transmission from a pregnant woman to the fetus via the placenta has been reported (CDC 2024; Khan et al. 2023; Mohamed et al. 2024).

The awareness and preparedness of healthcare professionals during epidemics play a vital role in safeguarding both individual and public health (Aynalem et al. 2024; Cipolletta et al. 2022; Koota et al. 2024; Morgan et al. 2022). Globally (Cipolletta et al. 2022; Koota et al. 2024; Morgan et al. 2022) and in Türkiye (Akkuş et al. 2022; Azizoğlu et al. 2022; Cihan et al. 2020; Yalman and Sancar 2021), nurses have been observed to be at the forefront of healthcare service delivery during the COVID‐19 pandemic, maintaining direct contact with infectious patients. Therefore, assessing the knowledge levels of nurses regarding Mpox and their perceptions of this disease is of strategic importance in strengthening epidemic preparedness efforts. Infectious disease outbreaks, such as Mpox, often lead to increased anxiety among healthcare workers due to uncertainties surrounding transmission risks, disease severity, and the effectiveness of preventive measures (Brooks et al. 2020; Garfin et al. 2020). Elevated anxiety levels during epidemics have been shown to impair healthcare workers' cognitive performance, decision‐making abilities, and emotional well‐being (Blakey and Abramowitz 2017; Mertens et al. 2020). Therefore, assessing both the knowledge and anxiety levels of nurses during emerging infectious disease outbreaks is critical for designing effective training, support strategies, and public health interventions (Aynalem et al. 2024; Orok et al. 2024). In the existing literature, several studies have investigated the knowledge and attitudes of healthcare workers toward Mpox (Amer et al. 2024; Aynalem et al. 2024; Jahromi et al. 2024; Khattab et al. 2024; Miraglia del Giudice et al. 2023; Orok et al. 2024). However, there is still a need for region‐specific data to tailor public health interventions effectively.

The Mpox guideline published by the Turkish Ministry of Health in 2022 was updated in August 2024 (T.C. Health Minister, n.d.). However, it is considered that this guide alone may not be sufficient for evaluating the level of knowledge and attitudes of healthcare workers. Therefore, the continuity and accessibility of training programs are essential (Azizoğlu et al. 2022; Boutros et al. 2023; Rascón et al. 2024). Although global literature (Amer et al. 2024; Aynalem et al. 2024; Jahromi et al. 2024) has expanded, no studies have been conducted in Türkiye examining the knowledge levels and concerns of nurses or other healthcare workers regarding Mpox disease. The results of this study are expected to provide valuable insights into the existing knowledge gaps, the concerns of nurses, and the development of appropriate training and support programs. Furthermore, the findings may contribute to guiding health policymakers and educators in enhancing the competencies of nurses for more effective epidemic response.

## 2 Study Aims

The aim of this study was to evaluate nurses' knowledge and anxiety levels regarding human monkeypox virus infection across various healthcare institutions in Türkiye.

## 3 Materials and Methods

### 3.1 Study Design

A descriptive and cross‐sectional design was employed in this study to collect data.

### 3.2 Study Sampling

Eligible nurses were recruited through an online survey distributed via the researchers' personal social media accounts. Nurses working in public or private hospitals, family health centers, and other primary healthcare institutions across Türkiye were invited to participate. Participants were encouraged to further share the survey link with professional contacts, family, and friends to enhance sample diversity.

Out of 398 nurses who initially responded, 184 were excluded based on predefined criteria: 88 had retired, 55 had been quarantined during the COVID‐19 pandemic, and 41 had experienced the loss of a family member due to COVID‐19. Participants with a history of quarantine or family loss due to COVID‐19 were excluded in order to minimize the potential confounding effects of prior epidemic‐related psychological distress and ensure a focused assessment of Mpox‐specific anxiety. This methodological approach aimed to enhance the validity of the findings. These exclusions were implemented to minimize the potential influence of psychological distress and to ensure that the sample reflected actively practicing nurses. As a result, the final sample included 214 participants.

A purposive sampling strategy was employed to recruit participants. The sample size was determined using the Slovin formula, based on the known population size (*N*) and an acceptable margin of error (*e*) (Nyimbili and Nyimbili 2024). Assuming a margin of error of 5% and a confidence level of 95%, the minimum required sample size was calculated as 133.33. The Slovin formula is expressed as:

$$n=\frac{N}{1+N{\mathrm{e}}^{2}}$$

The final sample size of 214 nurses exceeded the calculated minimum, ensuring statistical stability and reliability.

### 3.3 Procedures

Data collection was conducted through a self‐administered online questionnaire between 3 October 2024 and 3 December 2024. Participants accessed the survey via a secure link distributed on the researchers' personal social media accounts. Prior to participation, nurses were provided with an information sheet detailing the study's objectives, procedures, confidentiality assurances, and their right to withdraw at any time without penalty. Informed consent was obtained electronically before the participants proceeded to the survey.

The questionnaire was designed to be completed independently and was structured to prevent missing data, requiring all questions to be answered before submission. Detailed instructions were provided at the beginning of each section of the questionnaire to ensure clarity and facilitate accurate responses. Participant anonymity was maintained by avoiding the collection of any personally identifiable information. The platform's security protocols protected the confidentiality of all responses. Confidentiality was ensured by not collecting any personally identifiable information, and all responses were stored securely.

While the online method facilitated wide geographic participation and convenience, the potential for sampling bias due to the non‐random recruitment approach was acknowledged. In the first section of the online data collection form, participants were informed about the study, the purpose of the research was explained, and an electronic consent button was provided. Participants were assured that their responses would remain anonymous and that all collected data would be stored securely in password‐protected files accessible only to the research team and used solely for research purposes. Participants completed the survey in approximately 10–15 min. Participants did not receive any financial or material compensation for their involvement.

### 3.4 Measures

Nurse identification form consists of 17 questions about the demographic data of the nurses, including age, education, occupational information, and training related to Mpox.

### 3.5 Thoughts About Mpox

The form includes multiple‐choice questions based on the existing literature, assessing various dimensions of anxiety related to Mpox. The items address anxiety about contracting the disease, coping with death or the concept of death, uncertainty regarding treatment, anxiety about transmitting the disease to family, social environment, or workmates, fear of losing family members, friends, or colleagues due to the disease, anxiety about being unable to see family again, concerns related to vaccination, epidemic‐related anxiety, and the absence of any anxiety (Aynalem et al. 2024; Miraglia del Giudice et al. 2023; Mohamed et al. 2024; Sayar et al. 2020).

### 3.6 The FormNurse's Knowledge of Mpox

The Form was developed based on the existing literature regarding Mpox (Amer et al. 2024; Aynalem et al. 2024; Cipolletta et al. 2022; Miraglia del Giudice et al. 2023; Mohamed et al. 2024; Orok et al. 2024). It includes multiple‐choice questions designed to evaluate nurses' knowledge about the epidemiology, modes of transmission, clinical symptoms, prevention, and management strategies of Mpox. The questionnaire consists of 30 items, which nurses respond to as True, False, or Don't Know. These items are categorized under three sections: Knowledge (questions 1–16), Patient Control (questions 17–24), and Prevention (questions 25–30).

### 3.7 Epidemic Anxiety Scale (EAS)

The EAS is a measurement tool developed by Sayar et al. (2020) to assess anxiety levels related to epidemics. Following validity and reliability studies, the scale was finalized with 18 items grouped under four factors. The EAS is structured as a five‐point Likert‐type scale, with response options ranging from “Completely Suitable for Me,” “Very Suitable for Me,” “Moderately Suitable for Me,” “Less Suitable for Me,” to “Not Suitable for Me at All.” The total score ranges from 18 to 90, with higher scores indicating higher levels of epidemic anxiety. To interpret the total scores, a score between 18 and 32 indicates no anxiety, a score between 33 and 46 indicates little anxiety, a score between 47 and 61 indicates moderate anxiety, a score between 62 and 75 indicates high anxiety, and a score between 76 and 90 indicates very high anxiety (Sayar et al. 2020).

### 3.8 Statistical Analysis

Data analysis was performed using the SPSS 20.0 software (IBM SPSS Statistics for Windows, IBM Corp., Armonk, NY, USA). Descriptive statistics (number, percentage, mean, standard deviation, median) and frequency distributions were calculated for the study variables. The normality of continuous variables was tested using the Kolmogorov–Smirnov test. The direction and strength of relationships between independent variables and the number of correct responses on the EAS and Mpox knowledge assessments were determined using Pearson correlation analysis, one‐way ANOVA, and independent samples *t* test. One‐way ANOVA was applied to compare the mean scores between groups with more than two categories, such as educational status and unit of employment. Independent samples t‐test was used for comparisons between two groups, such as gender. This test was selected because it is appropriate for comparing means between two independent groups. Additionally, simple linear regression analysis was conducted to examine the effects of epidemic anxiety and demographic variables on knowledge scores. In this analysis, the dependent variable was the “number of correct responses on the Mpox knowledge test,” while the independent variable was “having sufficient information about the procedures implemented in their institutions regarding Mpox disease.”

A *p* value of ≤ 0.05 was considered statistically significant (Eroglu et al. 2013).

## 4 Results

This study is among the first studies conducted in Türkiye to evaluate nurses' levels of knowledge regarding infectious diseases such as Mpox. The findings indicate that the majority of participants had no prior experience providing care to patients diagnosed with Mpox and demonstrated insufficient knowledge about the disease.

Table 1 summarizes the socio‐demographic characteristics of the participants. The average age was 33.56 ± 9.81 years, with the majority being in the 20–29 age range. Most participants were male (85%) and held an undergraduate degree (66.4%). A large proportion (82.7%) worked as clinical nurses, and over half (51.9%) worked regular daytime shifts. While 70% expressed satisfaction with their profession, 66.8% stated they did not consider leaving nursing. Notably, nearly all participants (97.2%) had no prior experience caring for Mpox patients, and over 80% lacked institutional training or information about Mpox procedures.

**TABLE 1:** Socio‐demographic characteristics of the participants (*n* = 214).

Variables		*N* (%)
Age (mean = 33.56 years, SD = 9.81)	20–29 years	96 (44.9)
	30–39 years	52 (24.3)
	≥ 40 years	66 (30.8)
Gender	Woman	182 (85.0)
	Male	32 (15.0)
Marital status	Single	94 (43.9)
	Married	120 (56.1)
Education level	Health vocational high School	20 (9.3)
	Associate degree	14 (6.5)
	Undergraduate	142 (66.4)
	Postgraduate	38 (17.8)
Position in the unit	Nurse	177 (82.7)
	Infection nurse	4 (1.9)
	Education nurse	10 (4.7)
	Nurse manager	23 (10.7)
Mode of operation	Continuous daytime	111 (51.9)
	Shift Procedure	99 (46.3)
	Constantly at night	4 (1.9)
Weekly working hours	Good	70 (32.7)
	Fair	108 (50.5)
	Bad	36 (16.8)
Are you happy being a nurse?	Yes	150 (70.1)
	No	64 (29.9)
Are you thinking of leaving nursing?	Yes	71 (33.2)
	No	143 (66.8)
Have you cared for a patient diagnosed with Mpox?	Yes	6 (2.8)
	No	208 (97.2)
Do you have sufficient information about the procedures applied in your organization regarding Mpox disease?	Yes	33 (15.4)
	No	181 (84.6)
Are trainings on Mpox given in your organization?	Yes	35 (16.4)
	No	179 (83.6)
Do you attend trainings about Mpox?	Yes	27 (12.6)
	No	177 (87.4)
Duration of professional experience	Mean ± SD	11.66 (10.39)
Duration of experience in the organization	Mean ± SD	8.52 (9.04)

Table 2 summarizes the participants' fear‐related thoughts about Mpox. Over half of the participants (51.9%) reported fear of contracting the disease, and approximately one‐third expressed fear of transmitting it to others or losing loved ones. Additional concerns included anxiety about vaccination, treatment uncertainty, and epidemic‐related distress. Notably, 14.5% of participants reported no fear associated with Mpox.

**TABLE 2:** Thoughts of fear related to Mpox.

Variables		*N* = 214
Fear of contracting the disease	Yes	111 (51.9)
	No	103 (48.1)
Fear of dealing with death/dying	Yes	45 (21.0)
	No	169 (19.0)
Fear of uncertainty about treatment	Yes	100 (46.7)
	No	114 (53.3)
Fear of transmitting the disease to family/social environment/workmates	Yes	74 (34.6)
	No	140 (65.4)
Fear of losing family members/social environment/colleagues due to illness	Yes	66 (30.8)
	No	148 (69.2)
Fear of not seeing the family again	Yes	36 (16.8)
	No	178 (83.2)
Fear of vaccination	Yes	53 (24.8)
	No	161 (75.2)
Fear of pandemic	Yes	77 (36.0)
	No	137 (64.0)
I do not experience fear	Yes	31 (14.5)
	No	183 (85.5)

Table 3 presents the total and sub‐dimension scores of the EAS. The mean total EAS score of the participants was 48.36 ± 19.26. Regarding sub‐dimensions, the participants had a mean score of 17.67 ± 7.48 for the epidemic factor, 4.83 ± 2.40 for the economic factor, 11.40 ± 4.90 for the quarantine factor, and 14.45 ± 6.09 for the social life factor. Additionally, it was found that 28.5% of the participants were classified as moderately anxious according to the EAS total score interpretation.

**TABLE 3:** Epidemic disease anxiety scale scores.

Variable	*N*	%
Total points		
No anxiety	49	22.9
Less anxious	51	23.8
Medium anxious	61	28.5
Highly anxious	30	14.0
Very high anxiety	23	10.7

Factors	Mean	SD
Total points	48.36	19.26
Epidemic factor	17.67	7.48
Economic factor	4.83	2.40
Quarantine factor	11.40	4.90
Social life factor	14.45	6.09

**FIGURE 1:**
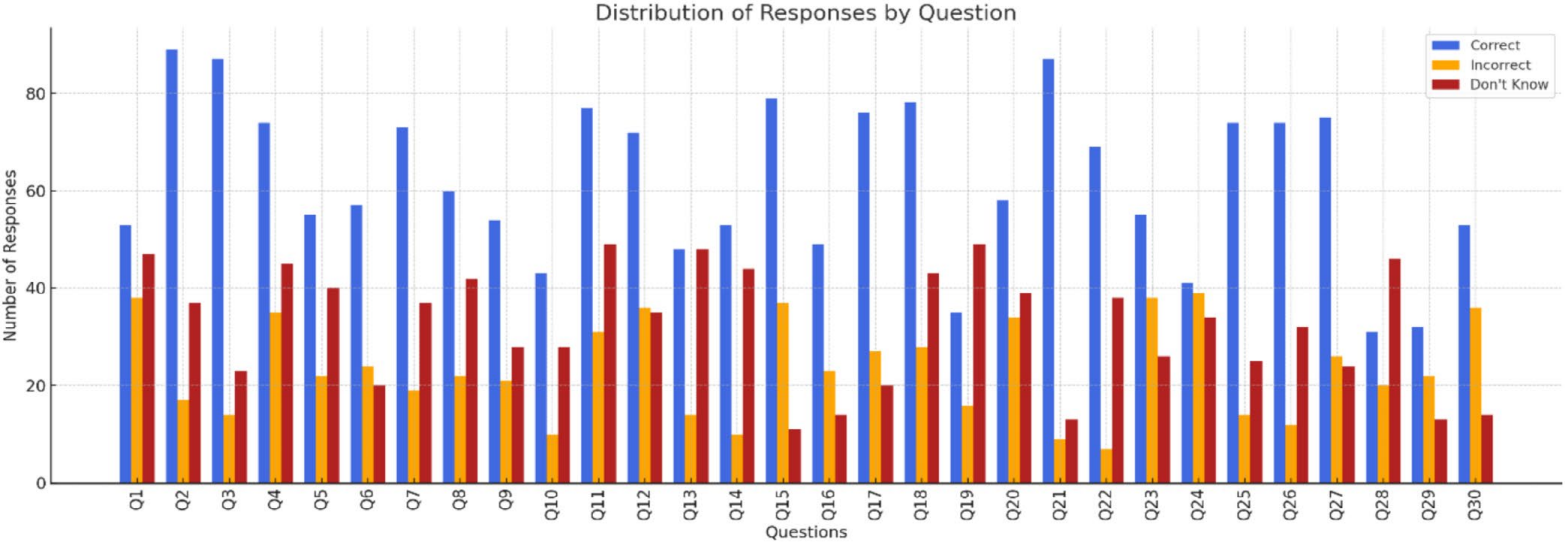
Ratios of correct, incorrect, and do not know answers of nurses' Mpox knowledge.

Figure 1 illustrates the distribution of responses to the Mpox knowledge questions. The highest correct response rate (83.6%) was observed for the item regarding transmission through close contact. In contrast, significant knowledge gaps were evident in questions related to ventilation requirements and post‐exposure vaccination, with high rates of incorrect or “don't know” responses. These findings suggest specific areas where educational efforts should be strengthened.

Table 4 shows the participants' performance on the Mpox knowledge assessment. The average number of correct answers was 16.96 ± 8.84 out of 26. Overall, knowledge levels varied considerably, with correct response rates ranging from 27.6% to 83.6%. Knowledge was highest in items related to symptoms and transmission, while prevention‐related items had the lowest accuracy rates.

**TABLE 4:** Correct answer rates of questions about Mpox knowledge (*n* = 214).

	Correct answer	*N* (%)
*Disease symptom and transmission information*		
1. Mpox is a zoonotic disease, it can spread between animals and humans	Yes	175 (81.8)
2. The virus is spread by close contact with a person with Mpox	Yes	179 (83.6)
3. The virus is spread by direct contact with contaminated materials	Yes	150 (70.1)
4. The incubation period is 3–17 days.	Yes	93 (43.5)
5. During the incubation period, a person has no symptoms and may feel well. During this time the person is not contagious.	No	148 (69.2)
6. The lesions are hard or rubbery, well circumscribed, deeply located and often have a apex.	Yes	114 (53.3)
7. Lesions often occur in the genital and anorectal areas or in the mouth.	Yes	83 (38.8)
8. The rash may be limited to only a few lesions or a single lesion.	Yes	80 (37.4)
9. The rash does not always appear on the palms of the hands and soles of the feet.	Yes	81 (37.9)
10. Fever and other prodromal symptoms (e.g., chills, lymphadenopathy, malaise, myalgia or headache) may or may not occur before or after the rash.	Yes	112 (52.3)
11. Respiratory symptoms (e.g., sore throat, nasal congestion or cough).	Yes	115 (53.7)
12. The evolution of the lesions passes through four stages: macular, papular, vesicular, pustular, macular, papular, vesicular, pustular before crusting and desquamation.	Yes	98 (45.8)
13. The person is contagious until all crusts on the skin have fallen off and a fresh layer of intact skin has formed underneath.	Yes	104 (48.6)
14. The disease typically lasts 2–4 weeks.	Yes	90 (42.1)
15. The severity of the disease depends on the individual's state of health and the route of exposure.	Yes	131 (61.2)
16. Health personnel should be monitored for up to 21 days after exposure.	Yes	98 (45.8)
*Patient control information*		
17. The patient should be kept in an isolated room	Yes	164 (76.6)
18. Procedures that may reactivate dried material from lesions (e.g., use of portable fans, dry dusting, sweeping, vacuuming) should be avoided.	Yes	111 (51.9)
19. No special ventilation is required.	No	97 (45.3)
20. The door must be kept closed	Yes	113 (52.8)
21. The patient must have a private bathroom	Yes	156 (72.9)
22. If transported outside the patient's room, a careful source check (e.g., medical mask) should be carried out.	Yes	149 (69.6)
23. If the patient is moved outside the room, cover exposed skin lesions with a sheet or apron.	Yes	134 (62.6)
24. Protective equipment apron, goggles, gloves and N95 mask should be used when entering the patient room.	Yes	160 (74.8)
*Prevention information*		
25. Close skin‐to‐skin contact with people with a rash that looks like Mpox and with animals infected with Mpox virus should be avoided.	Yes	159 (74.3)
26. You need infection control steps to reduce your risk of Mpox during sex or at a social gathering	Yes	150 (70.1)
27. Vaccination	Yes	89 (41.6)
28. People at risk of Mpox should ideally be vaccinated pre‐exposure.	Yes	96 (44.9)
29. Vaccination is also required after exposure to the virus.	Yes	59 (27.6)
30. People with a weak immune system, children younger than 1 year, people with a history of eczema, pregnant women are at risk.	Yes	135 (63.1)
Mean number of correct: Mean ± SD = 16.96 ± 8.84		

Table 5 presents the relationship between EAS scores and Mpox knowledge, evaluated using the Pearson correlation test. The analysis revealed a positive correlation between EAS scores and Mpox knowledge scores. Specifically, an increase in the number of correct answers on the Mpox knowledge test was associated with higher EAS scores.

**TABLE 5:** Relationship between EAS and Mpox knowledge.

Variable	*N*	*r*	*p*
EAS total score	214	0.2	0.003*
Number of correct answers in Mpox information			

Abbreviation: *r* = correlation test.

* *p* ≤ 0.05.

Table 6 presents the comparison of the total Epidemic Anxiety Scale (EAS) scores and the number of correct responses on the Mpox knowledge test according to the socio‐demographic characteristics of the participants. One‐way ANOVA, Pearson correlation analysis, and independent samples t‐tests were applied for the analyses. A statistically significant difference was found in EAS total scores according to the gender variable (*p* ≤ 0.05), with female participants having higher mean scores than male participants. Additionally, a significant difference in the number of correct answers on the Mpox knowledge test was observed across age groups, satisfaction with being a nurse, having sufficient information about institutional procedures regarding Mpox disease, availability of Mpox‐related trainings in the institution, and participation in Mpox‐related trainings (*p* ≤ 0.05). Although a significant difference was detected according to age groups, no statistically significant differences were found between the specific groups in the post hoc Bonferroni test.

**TABLE 6:** Comparison of the total score of the EAS and the number of correct answers in Mpox information according to the socio‐demographic characteristics of the participants.

Variable (*n* = 214)		Total score of SCA		Mpox information number of correct answers	
		Test value	*p*	Test value	*p*
Age	20–29 years	*F* = 0.138	0.871	*F* = 3.192	0.043*
	30–39 years				
	≥ 40 years				
Gender	Woman (1)	*t* = 2.612	0.01* (1 > 2)	*t* = −0.182	0.856
	Male (2)				
Marital status	Single	*t* = 1.310	0.192	*t* = −1.658	0.099
	Married				
Education level	High school	*F* = 0.449	0.718	*F* = 0.313	0.816
	Associate degree				
	Undergraduate				
	Postgraduate				
Position in the unit	Nurse	*F* = 1.691	0.170	*F* = 0.978	0.404
	Infection nurse				
	Education nurse				
	Nurse manager				
Mode of operation	Continuous daytime	*F* = 0.740	0.478	*F* = 0.671	0.512
	Shift procedure				
	Constantly at night				
Weekly working hours	Good	*F* = 1.023	0.361	*F* = 2.374	0.096
	Fair				
	Bad				
Are you happy being a nurse?	Yes (1)	*t* = −1.568	0.118	*t* = 2.034	0.043* (1 > 2)
	No (2)				
Are you thinking of leaving nursing?	Yes	*t* = 1.650	0.100	*t* = −1.296	0.196
	No				
Have you cared for a patient diagnosed with Mpox?	Yes	*t* = −1.382	0.168	*t* = 0.770	0.442
	No				
Do you have sufficient information about the procedures applied in your institution regarding Mpox disease?	Yes (1)	*t* = 0.402	0.688	*t* = 4.083	0.000* (1 > 2)
	No (2)				
Does your organization provide trainings on Mpox?	Yes (1)	*t* = 0.108	0.914	*t* = 3.678	0.000* (1 > 2)
	No (2)				
Do you attend Mpox related trainings?	Yes (1)	*t* = −0.318	0.751	*t* = 2.520	0.012* (1 > 2)
	No (2)				
Duration of professional experience		*r* = 0.046	0.500	*r* = 0.098	0.153
Duration of experience in the organization		*r* = 0.092	0.186	*r* = 0.092	0.189

Abbreviations: *F* = ANOVA test; *r* = Pearson correlation test; *t* = independent *t* test.

* *p* ≤ 0.05.

Participants who reported satisfaction with being a nurse, those who had sufficient information about institutional Mpox procedures, those whose institutions provided Mpox‐related training, and those who participated in Mpox‐related training demonstrated higher Mpox knowledge scores compared to their counterparts.

Table 7 presents the comparison of the total Epidemic Anxiety Scale (EAS) scores and the number of correct responses on the Mpox knowledge test according to participants' fear‐related thoughts about Mpox. Independent samples t‐test was applied for the analysis. Statistically significant differences were found for all variables (*p* ≤ 0.05). Specifically, significant differences in the number of correct answers on the Mpox knowledge test were observed based on participants' fear of contracting the disease, fear of transmitting the disease to family, social environment, or colleagues, fear of losing family members, friends, or colleagues due to the disease, fear of not seeing family again, fear associated with epidemic‐related anxiety, and absence of fear.

**TABLE 7:** Comparison of the EAS total score and the number of correct answers in Mpox information according to the participants' fear of Mpox.

Variable (*n* = 214)		Total score of SCA		Mpox knowledge level number of correct answers	
		Test value	*p*	Test value	*p*
Fear of contracting the disease	Yes	*t* = 4.295	0.000* (1 > 2)	*t* = 3.239	0.001* (1 > 2)
	No				
Fear of dealing with death/dying	Yes	*t* = 3.793	0.000* (1 > 2)	*t* = 1.400	0.163
	No				
Fear of uncertainty about treatment	Yes	*t* = 3.453	0.001* (1 > 2)	*t* = 1.974	0.050
	No				
Fear of transmitting the disease to family/social environment/workmates	Yes	*t* = 5.781	0.000* (1 > 2)	*t* = 2.107	0.036*
	No				
Fear of losing family members/social environment/colleagues due to illness	Yes	*t* = 5.126	0.000* (1 > 2)	*t* = 2.562	0.011* (1 > 2)
	No				
Fear of not seeing the family again	Yes	*t* = 4.625	0.000* (1 > 2)	*t* = 2.417	0.016* (1 > 2)
	No				
Fear of vaccination	Yes	*t* = 3.479	0.001* (1 > 2)	*t* = 1.004	0.317
	No				
Pandemic fears	Yes	*t* = 3.154	0.002* (1 > 2)	*t* = 2.658	0.008* (1 > 2)
	No				
I do not experience fear	Yes	*t* = −4.800	0.000* (2 > 1)	*t* = −2.592	0.010* (2 > 1)
	No				

*Note:* 1: yes, 2: no.

Abbreviation: *t* = independent *t* test.

* *p* ≤ 0.05.

Table 8 presents the results of the simple linear regression analysis. The model was found to be statistically significant (*p* ≤ 0.05), indicating a meaningful relationship between the predictor and the outcome variable. Specifically, having sufficient information about institutional Mpox‐related procedures significantly predicted higher knowledge scores. The model accounted for 7.2% of the variance in Mpox knowledge scores, and each one‐unit increase in perceived sufficiency of information was associated with a 6.56 point increase in the number of correct answers.

**TABLE 8:** Regression analysis results.

Independent variable	Dependent variable	*β*	SE	Std *β*	*t*	*p*	*F*	Model (*p*)	*R* ^2^	DW
To have sufficient knowledge about the procedures related to Mpox disease in their institutions	Number of Mpox test correct answers	6.560	1.620	0.269	4.050	0.000	16.401	0.000*	0.072	1.920

Abbreviations: *β*: unstandardised coefficient; DW: Durbin–Watson statistic; *F*: *F* test value; Model (*p*): model significance level; *p*: *p* value; *R* ^2^: coefficient of determination; SE: standard error; Std *β*: standardized coefficient; *t*: *t* value.

* *p* ≤ 0.05.

## 5 Discussion

This study evaluated nurses' fears related to a new epidemic, their perceptions, and their knowledge levels regarding Mpox. In total, 51.9% of the participants reported fear of contracting the disease. Similar patterns of fear have been documented in previous outbreaks, such as the 2009–2010 H1N1 influenza pandemic and the 2015–2016 Zika virus outbreak, where concerns about infectiousness and disease severity were heightened (Brooks et al. 2020). During the COVID‐19 pandemic, Mertens et al. (2020) reported that 53.8% of participants experienced fear of infection, although it is important to recognize that the transmission dynamics and public health impact of COVID‐19 and Mpox differ (Mertens et al. 2020). Furthermore, Rony et al. (2023) highlighted that lack of sufficient knowledge was a major contributor to anxiety and fear during epidemic situations. In line with previous findings, the literature emphasizes that risk perception, knowledge level, and media exposure significantly influence fear responses during epidemics (Aljamaan et al. 2022; Zhang et al. 2021). In the present study, 30.8% of participants expressed fear of losing loved ones, a concern also prominently observed during the COVID‐19 pandemic (Garfin et al. 2020). Supporting these observations, Aljamaan et al. (2022) found that 49.6% of healthcare workers in Saudi Arabia expressed fear of contracting Mpox (Aljamaan et al. 2022), aligning closely with the results of the current study.

In the present study, 19.0% of participants reported fear due to uncertainty about treatment and 24.8% expressed concerns about vaccination. Although the rates in our study were lower, similar concerns were also reported by Amer et al. (2024). In their study, 45.8% of participants were uncertain about vaccine effectiveness, and 55.5% expressed concerns about vaccine safety. These findings suggest a common pattern of anxiety regarding Mpox prevention and management among healthcare professionals (Orok et al. 2024). In a similar vein, Zhang et al. (2021) reported that vaccine hesitancy during the COVID‐19 pandemic was significantly influenced by misinformation spread through social media. Nevertheless, it should be noted that the epidemiological characteristics of COVID‐19 and Mpox differ substantially (Zhang et al. 2021). Bates and Grijalva (2022) found that 51.7% of individuals were more likely to consider smallpox vaccination when they perceived themselves to be at greater risk of Mpox infection (*p* < 0.001) (Bates and Grijalva 2022). These findings highlight the role of perceived risk as a key determinant of vaccination behavior, particularly among vulnerable populations such as older adults and individuals with chronic conditions (Aljamaan et al. 2022). Accordingly, improving public knowledge through accurate and targeted information strategies is essential to reducing vaccine hesitancy and enhancing outbreak response.

### 5.1 Epidemic Anxiety

In the present study, the mean score obtained from the epidemic factor of the EAS was 17.67 ± 7.48. Comparatively, Kızılkaya and Çağatay (2023) reported a mean score of 16.71 ± 6.42 among healthcare professionals, while Kocakaya and Harmancı (2022) found a lower mean score of 13.04 ± 5.37 among students. Similarly, Aynalem et al. (2024) reported that anxiety levels among healthcare workers were significantly elevated during the Mpox outbreak, particularly in relation to epidemic‐specific stressors (Aynalem et al. 2024). They further noted that 62.5% of healthcare workers experienced fear of infection, which negatively impacted their work performance. In a similar vein, Orok et al. (2024) found that health professionals in Nigeria exhibited high levels of anxiety during the Mpox epidemic, which severely disrupted the delivery of health services (Orok et al. 2024). Such elevated anxiety levels among healthcare workers may partially explain the higher epidemic anxiety scores observed among nurses in the present study.

The elevated anxiety levels among nurses observed in this study may be attributable to their direct exposure to infection risks during epidemic periods, as supported by previous literature (Kızılkaya and Çağatay 2023). In addition to epidemic‐specific anxieties, economic concerns also emerged as a notable source of distress. In the present study, the mean score for the economic factor of the EAS was 4.83 ± 2.40, compared to 5.10 ± 2.21 reported by Kızılkaya and Çağatay (2023) and 4.72 ± 2.20 by Kocakaya and Harmancı (2022). Supporting these findings, Khattab et al. (2024) emphasized that economic factors during the Mpox outbreak adversely impacted both the professional motivation and overall well‐being of healthcare workers (Khattab et al. 2024). These results suggest that the economic anxiety scores observed in our study may reflect not only individual concerns but also the broader professional and social challenges faced by healthcare workers during epidemic periods.

In relation to the quarantine‐related subdimension of the EAS, the mean score in our study was 11.40 ± 4.90, compared to 10.46 ± 4.13 reported by Kızılkaya and Çağatay (2023) and 11.78 ± 3.88 by Kocakaya and Harmancı (2022). Supporting these findings, Torales et al. (2024) emphasized that young individuals are particularly vulnerable to the psychological effects of quarantine and isolation, which can significantly impact their social and emotional development (Torales et al. 2024). Furthermore, Brooks et al. (2020) highlighted that healthcare workers experienced increased anxiety during quarantine periods due to isolation from their social environments and families (Brooks et al. 2020). These findings collectively suggest that quarantine constitutes a major psychosocial stressor during epidemics, affecting not only healthcare workers but also the broader population. Therefore, developing support programs to manage quarantine‐related anxiety and to enhance both psychological and professional resilience among healthcare workers should be considered a key component of epidemic management strategies.

Regarding the social life subdimension of the EAS, the mean score obtained in our study was 14.45 ± 6.09. Similarly, Kızılkaya and Çağatay (2023) reported a mean score of 13.46 ± 5.59, and Kocakaya and Harmancı (2022) reported a mean score of 14.22 ± 4.84. The consistency of social life anxiety levels between healthcare professionals and students highlights that the restriction of social ties is a pervasive source of stress during epidemic periods. Particularly among healthcare workers, limited access to social support mechanisms due to professional responsibilities may exacerbate this anxiety. These observations align with previous findings that social isolation during epidemics not only affects individual psychosocial health but also has broader societal implications (Brooks et al. 2020).

In the present study, the total EAS score, encompassing the epidemic, economic, quarantine, and social life subdimensions, was 48.36 ± 19.26. Similarly, Kızılkaya and Çağatay (2023) reported a total score of 47.73 ± 1.66, while Kocakaya and Harmancı (2022) found a score of 43.88 ± 12.87. Consistent with these findings, Walsh‐Buhi et al. (2024) reported that individual perceptions and uncertainties regarding the Mpox outbreak significantly contributed to increased anxiety levels across a national sample in the United States. The elevated scores observed across the EAS subdimensions suggest that multiple factors, including fear of infection, social isolation, and economic concerns, collectively influenced the anxiety levels of nurses.

### 5.2 Relationship Between Mpox Knowledge and Epidemic Anxiety

The mean number of correct answers concerning Mpox knowledge was found to be 16.96 ± 8.84, indicating a moderate overall level of knowledge among participants. Correct response rates showed considerable variability across knowledge domains, with relatively higher accuracy observed for disease symptoms and transmission information, and lower accuracy for prevention‐related knowledge. In comparison, Amer et al. (2024) reported overall correct answer rates ranging from 8.7% to 88%, with relatively stronger knowledge in transmission but lower knowledge regarding prevention practices. Similarly, Miraglia del Giudice et al. (2023) found correct response rates between 5% and 75.8%, with especially low levels of prevention knowledge (4%–12.8%) (Miraglia del Giudice et al. 2023). Aynalem et al. (2024) reported correct response rates ranging from 37% to 80%, again highlighting moderate levels of knowledge on symptoms and transmission but notable gaps in prevention knowledge (Aynalem et al. 2024). Although the present study demonstrated somewhat better patient management and prevention knowledge compared to previous studies, the findings collectively point to persistent deficiencies in healthcare workers' understanding of prevention strategies and transmission routes. These results reinforce the necessity of targeted educational programs focusing on improving preventive practices, patient management strategies, and transmission control among nurses.

In the present study, a positive correlation was observed between Mpox knowledge and epidemic anxiety, indicating that increased knowledge levels were associated with heightened risk perception and anxiety. This pattern is consistent with the findings of Bates and Grijalva (2022), who reported that greater knowledge among healthcare workers about infections was associated with elevated anxiety levels (Bates and Grijalva 2022). Similarly, Islam et al. (2023) found that individuals with more extensive knowledge about infectious diseases such as Mpox exhibited greater concern regarding the contagiousness and potential impacts of the disease (Islam et al. 2023). These findings suggest that as individuals become more informed, their awareness of risks increases, which in turn may intensify anxiety. Supporting this perspective, a systematic review by Alhumaid et al. (2021) emphasized the need for effective and comprehensive training programs that not only enhance knowledge but also address the psychological aspects of risk perception anxiety (Alhumaid et al. 2021). Accordingly, educational interventions should be designed not only to improve factual knowledge but also to equip healthcare workers with strategies for managing anxiety related to perceived risks.

In the present study, female participants exhibited significantly higher total EAS scores related to Mpox compared to their male counterparts, consistent with existing literature indicating that women generally have higher risk perceptions during epidemics (Medina et al. 2022; Farhane‐Medina et al. 2022; Walsh‐Buhi et al. 2024). Neurobiological factors, such as differences in brain structure and the effects of fluctuating sex hormones, have been associated with heightened anxiety levels among women (Medina et al. 2022). Additionally, biopsychosocial factors contribute to increased anxiety and stress levels in women compared to men (Farhane‐Medina et al. 2022). Walsh‐Buhi et al. (2024) also reported that women experienced greater fear related to Mpox, supporting the findings of the present study. Moreover, the predominance of women in the nursing profession (85.0% in this study) and their greater emotional workload and exposure to infectious disease risks may further amplify anxiety levels among female nurses (Morgan et al. 2022). Although a difference in anxiety scores across age groups was observed in the present study, post hoc analysis revealed that this difference was not statistically significant, suggesting that age may be a less influential factor compared to individual knowledge levels. This finding aligns with previous studies indicating that experience and education, which tend to increase with age, can mitigate risk perception (Cihan et al. 2020). Furthermore, access to reliable information and educational resources has been shown to play a critical role in shaping individuals' risk perception and knowledge during pandemics (Cipolletta et al. 2022).

In the present study, individuals who were knowledgeable about institutional procedures related to Mpox demonstrated significantly higher knowledge accuracy, highlighting the critical role of access to information and training. This finding aligns with previous research emphasizing the positive impact of occupational satisfaction on job performance and the effective use of knowledge (Kato et al. 2023). Furthermore, the significant differences observed in Mpox knowledge scores across variables related to professional satisfaction and educational exposure support the assertion that access to training and increased job satisfaction enhance healthcare workers' knowledge and promote more informed behaviors (Koota et al. 2024). Specifically, nurses who reported being satisfied with their profession exhibited higher levels of Mpox knowledge, suggesting that professional satisfaction may improve motivation, attention, and the ability to utilize information effectively. Consistent with earlier findings, the present study also showed that participation in Mpox‐related training programs contributed to improved knowledge levels, echoing the conclusions of Boutros et al. (2023), Kaveh et al. (2022), Knutsen Glette et al. (2023), and Frenk et al. (2022) regarding the effectiveness of targeted educational interventions during pandemics. These findings underscore the importance of strengthening access to professional knowledge and structured training processes, particularly in contexts where healthcare workers face unclear workflows, a lack of standardized guidelines, and increased workloads (Knutsen Glette et al. 2023; Rascón et al. 2024; Millroth et al. 2021). Enhancing training opportunities and clear procedural frameworks appears to be a crucial element in improving pandemic preparedness and response among healthcare professionals.

In the present study, significant differences were observed between participants' Mpox knowledge accuracy, epidemic anxiety scores, and their reported fears regarding Mpox, indicating that risk perception and uncertainty contribute substantially to elevated anxiety levels. This finding is consistent with previous research by Campolino et al. (2022), who demonstrated that both education level and quality of information are closely associated with risk perception. Similarly, Millroth and Frey (2021) emphasized that individuals with higher aversion to risk and intolerance to uncertainty, particularly those influenced by prior negative experiences, are more prone to heightened fear and anxiety. Taken together, these findings suggest that heightened fear levels not only exacerbate anxiety but may also impair cognitive functioning, leading to difficulties in concentration, impaired information processing, and weakened critical thinking skills. In the context of healthcare workers, particularly nurses, fear of losing loved ones during an Mpox outbreak may disrupt their ability to acquire and apply accurate information effectively. Further supporting this, Beckers et al. (2023) reported that elevated fear levels directly impair psychological well‐being. Similarly, Kıssal and Akyazı (2024) and Koçak et al. (2021) emphasized that specific fears, including fear of contracting the disease, fear of transmitting it to family and colleagues, and fear related to pandemics, are critical determinants of anxiety levels. In addition, Rodríguez‐Rey et al. (2020) highlighted that anxiety during pandemics is shaped not only by health‐related risks but also by broader social stressors, such as having loved ones infected, shortages of essential supplies, dissatisfaction with governmental response, and uncertainty about the duration of the crisis. Collectively, these findings indicate that unmanaged fear and uncertainty may not only elevate anxiety but also compromise cognitive performance, decision‐making, and information processing abilities among healthcare workers. Therefore, interventions designed to address both informational needs and psychological resilience are essential to ensure the cognitive and emotional readiness of healthcare professionals during public health emergencies.

In the present study, it was observed that participants who reported no significant fears regarding Mpox exhibited lower anxiety levels and provided answers that were more accurate in relation to Mpox knowledge. This finding suggests that fear may interfere with cognitive processing and knowledge acquisition during outbreaks.

Interestingly, Zeidan et al. (2023) reported that individuals with a higher level of Mpox knowledge exhibited higher anxiety levels, suggesting that increased awareness may heighten perceived threat and psychological distress. In contrast, Mohammad et al. (2024) reported that among nursing students, higher levels of Mpox knowledge were positively associated with more favorable attitudes and negatively associated with anxiety and perceived mental health needs. Their findings suggest that educational interventions targeting neutral or negative attitudes could contribute to improving knowledge levels and reducing anxiety. Taken together, these findings highlight that while knowledge acquisition is critical for preparedness, it must be accompanied by psychological support strategies to manage fear and threat perceptions. Consistent with the literature, the present study also found that having sufficient knowledge about institutional Mpox‐related procedures significantly improved the accuracy of Mpox knowledge responses, aligning with previous reports that emphasized the role of education and training in enhancing healthcare workers' competence (Amer et al. 2024; Boutros et al. 2023; Rascón et al. 2024). Similarly, Ntawuyamara et al. (2025) reported that Burundian healthcare workers exhibited low levels of Mpox knowledge and lacked confidence in diagnosis, treatment, and prevention, highlighting the urgent need for continuous medical education in Mpox epidemiology and outbreak preparedness.

In the current study, it was observed that 7.2% of the variance in the number of correct Mpox knowledge responses could be explained by participants' knowledge of institutional procedures. Although this percentage is modest, it underscores the importance of systematic educational interventions. Considering the mean number of correct responses (16.96 ± 8.84), it appears crucial to urgently implement targeted nurse education programs on Mpox in Türkiye, similar to international efforts such as those recommended for Burundian health workers (Ntawuyamara et al. 2025).

## 6 Limitation and Recommendations

This study has several limitations that should be considered when interpreting the findings. Additionally, although the study was conducted across Türkiye, regional differences in healthcare infrastructure, training accessibility, and exposure to global travel may influence Mpox preparedness levels. Therefore, the findings should be interpreted considering Türkiye's diverse healthcare landscape and its susceptibility to infectious disease outbreaks due to high rates of international tourism and student mobility. First, the cross‐sectional design of the study limits causal inferences, as it captures only a snapshot of the participants' knowledge and anxiety levels at a single point in time. Second, data were collected through self‐reported questionnaires, which may introduce reporting biases or inaccuracies due to participants' subjective perceptions. Third, nurses who experienced quarantine or family loss during the COVID‐19 pandemic were excluded from the study, which may restrict the generalizability of the results. These excluded participants may have had distinct psychological profiles or heightened anxiety responses compared to the included participants, potentially influencing the overall findings. Future studies could benefit from adopting longitudinal designs to assess changes over time and to better establish causal relationships. In addition, incorporating objective measures alongside self‐report instruments and including more diverse nursing populations would enhance the robustness and generalizability of the results.

## 7 Conclusions

Although only one confirmed case of Mpox has been reported in Türkiye to date, the country remains at significant risk due to its extensive international tourism, student mobility, and global interconnectedness. Given the zoonotic nature of Mpox and its potential for cross‐border transmission, proactive educational interventions are necessary to ensure that healthcare personnel are adequately prepared for potential future outbreaks.

This study is the first in Türkiye to assess the awareness and educational needs of nurses regarding Mpox. The findings reveal that most participants had neither provided care for patients diagnosed with Mpox nor received adequate training on Mpox‐related procedures within their institutions. The lack of knowledge among nurses may hinder effective patient care and infection control efforts during potential outbreaks.

These results highlight the urgent need to implement structured and standardized educational programs focusing on emerging infectious diseases such as Mpox. Prioritizing infectious disease training within healthcare institutions will not only contribute to the professional development of nurses but also strengthen the overall quality and safety of healthcare services.

From a health policy perspective, it is recommended to mandate infectious disease training programs, integrate outbreak management modules into nursing curricula, and establish national guidelines for epidemic preparedness. Strengthening the educational infrastructure for healthcare workers will play a critical role in enhancing Türkiye's public health resilience against emerging global health threats.

### 7.1 Relevance for Clinical Practice

This study highlights critical gaps in nurses' knowledge and anxiety management regarding Mpox in Türkiye. While Türkiye has reported only a single confirmed case, the country's high levels of international travel and student mobility create an ongoing risk for future outbreaks. Proactive assessment of healthcare workers' preparedness is therefore crucial. Understanding these gaps enables the development of evidence‐based training programs that not only enhance epidemic readiness but also protect patient safety and ensure rapid clinical response during public health emergencies.

## Author Contributions

**Yasemin Karacan:** conceptualization, formal analysis, investigation, methodology, supervision, writing original draft, writing review and editing. **Rıdvan Bayram:** conceptualization, data curation, writing original draft, writing review and editing. **Serkan Budak:** conceptualization, formal analysis, investigation, methodology, supervision, writing original draft, writing review and editing.

## Ethics Statement

To conduct the study, ethical approval dated 2 October 2024 and numbered 220 was obtained from the Non‐Interventional Clinical Research Ethics Committee of Yalova University, where the research was conducted. The study was performed in accordance with the principles of the Declaration of Helsinki.

## Consent

Eligible nurses actively working in public and private hospitals, family health centers, and other primary healthcare institutions across Türkiye participated voluntarily in this study. Data were collected through a self‐administered online questionnaire designed to assess their knowledge and concerns regarding Mpox. Participants were informed about the study's purpose and procedures before providing their responses.

## Conflicts of Interest

The authors declare no conflicts of interest.

## Supporting information


**Data S1.** STROBE Statement—Checklist of items that should be included in reports of cross‐sectional studies.

## Data Availability

The data that support the findings of this study are available from the corresponding author upon reasonable request.
